# Psychometric evaluation of the Brazilian version of the Postpartum Specific Anxiety Scale – Research Short-Form for Global Crises (PSAS-BR-RSF-C)

**DOI:** 10.1007/s00737-026-01771-6

**Published:** 2026-09-26

**Authors:** Bruna Cardoso Gerhardt, Camila Zimmer, Jovana Serra, Kydja M. Souza Torres de Araújo, Victoria Fallon, Sergio A. Silverio, Adriane Xavier Arteche

**Affiliations:** 1 Pontifical Catholic University of Rio Grande do Sul, Porto Alegre, Brazil; 2 Federal University of Pernambuco, Recife, Brazil; 3 University of Liverpool, Liverpool, UK; 4 King’s College London, London, UK

**Keywords:** Postpartum anxiety, Psychometrics, Validation, Pandemic, Health system shock

## Abstract

**Purpose:**

Anxiety symptoms are highly prevalent during the postpartum period; however, validated instruments to measure postpartum-specific anxiety in Brazil are lacking. This study aimed to evaluate the psychometric properties of the Brazilian Portuguese version of the Postpartum Specific Anxiety Scale – Research Short-Form for Global Crises (PSAS-BR-RSF-C) in a Brazilian sample.

**Methods:**

A convenience sample of 189 Brazilian mothers of infants aged 0 to 12 months completed the PSAS-BR-RSF-C, the Edinburgh Postnatal Depression Scale (EPDS), the Generalized Anxiety Disorder scale (GAD-7), and the Self-Compassion Scale (SCS) online. Spearman correlations, confirmatory factor analysis (CFA) with Diagonally Weighted Least Squares estimation, and McDonald’s omega reliability indices were calculated.

**Results:**

CFA supported the four-factor structure proposed for the original instrument (CFI = 0.978; TLI = 0.970; RMSEA = 0.060 [90% CI: 0.035–0.083]; SRMR = 0.077). The PSAS-BR-RSF-C total score showed moderate-to-strong and significant correlations with measures of anxiety (GAD-7; *r* = .52), depression (EPDS; *r* = .61), and self-criticism (SCS; *r* = .52), demonstrating good convergent validity. Overall internal consistency was satisfactory (McDonald’s ω = 0.85).

**Conclusion:**

These findings provide initial support for the validity and overall internal consistency of the PSAS-BR-RSF-C in Brazilian postpartum samples, while indicating that the reliability of some factor scores warrants further investigation. The instrument offers a brief, culturally adapted tool for researchers and clinicians assessing postpartum anxiety in Brazil, including during global health crises.

**Supplementary Information:**

The online version contains supplementary material available at https://doi.org/10.1007/s00737-026-01771-6.

## Article highlights

- The PSAS-BR-RSF-C provides a brief measure of postpartum-specific anxiety for Brazilian mothers.
- A four-factor structure with good fit was confirmed via confirmatory factor analysis.
- Moderate-to-strong convergent validity was found with anxiety, depression, and self-criticism measures.
- The scale is brief (12 items) and suitable for use during global health crises.
- Future research should test the scale across diverse socioeconomic groups in Brazil.

**Supplementary Information:**

The online version contains supplementary material available at https://doi.org/10.1007/s00737-026-01771-6.

## Introduction

Perinatal mental health is a critical public health concern due to its association with a wide range of adverse outcomes for both mothers and infants (Vigod et al. 2025). Anxiety disorders are highly prevalent during pregnancy and the postpartum period, yet they remain underdiagnosed and undertreated, partly because of the lack of specific assessment tools (Dennis et al. 2017; Fallon et al. 2016a, b). Most available measures were developed for the general population, potentially overlooking key aspects of postpartum anxiety. In Brazil, the assessment of postpartum-specific anxiety remains limited, highlighting the need to expand the availability of culturally adapted and psychometrically sound measures.

In low- and middle-income countries, anxiety symptoms impact 29.2% of women during pregnancy and 24.4% during the postpartum period (Nielsen-Scott et al. 2022), with diagnosed anxiety disorders affecting 8.1% and 16% of mothers, respectively. In Brazil, a cohort study with follow-up during the COVID-19 pandemic revealed that 25.9% of 1,028 women assessed met the criteria for probable generalized anxiety disorder (GAD) based on the GAD-7 scale (Loret de Mola et al. 2021). This prevalence was 2.4 times higher than that reported at baseline in 2019, prior to the pandemic.

Despite greater focus on postpartum depression, it is widely accepted that postpartum anxiety can also present complications in perinatal populations for both the mother and the baby (Domínguez-Solís et al. 2021) and is likely to occur at higher rates than postpartum depression. High maternal anxiety levels are linked to lower maternal self-efficacy, impaired bonding (Goodman et al. 2016), reduced breastfeeding rates (Fallon et al. 2016a, b), and an increased risk of postpartum depression (Guvenc et al. 2021; Teissedre and Chabol 2003). Furthermore, maternal anxiety is associated with developmental delays, behavioral problems (Mughal et al. 2019; Field 2018), and poorer overall child outcomes (Glasheen et al. 2010), underscoring the need for early detection and intervention.

However, there is no consensus on how to measure postpartum anxiety (Harris et al. 2024; Worrall et al. 2023). Research often relies on anxiety scales designed for the general population, which may not fully capture the unique features of postpartum anxiety (Matthey et al. 2003; Silverio et al. 2021). Generic measures tend to include symptoms common in the perinatal period — such as fatigue and irritability — but may overlook excessive worries about the baby and maternal role (Fallon et al. 2016a, b). Additionally, symptom overlap with postpartum depression measures can lead to misclassification and underreporting of anxiety cases (Loyal et al. 2020; Fallon et al. 2016a, b; Goodman et al. 2016). While postpartum anxiety lacks formal diagnostic criteria, it is widely recognized as a distinct phenomenon influenced by the transition to motherhood and maternal–infant interactions (Dennis and Falah-Hassani 2017).

The Postpartum Specific Anxiety Scale (PSAS) was developed to address the lack of specific measures for assessing postpartum anxiety (Fallon et al. 2016a, b). This 51-item scale is structured into four factors capturing key aspects of postpartum anxiety, including maternal self-efficacy, mother–infant bonding, infant health and routine concerns, and broader psychosocial stressors (Davies et al. 2021). To improve feasibility, a research short-form (PSAS-RSF) (Davies et al. 2021) and an adapted version for use during global crises (PSAS-RSF-C) (Silverio et al. 2021) were later introduced and validated for English-speaking populations up to 12 months postpartum.

Recognizing the need for cross-cultural applicability, validated translations of the PSAS have been developed in multiple languages, including Brazilian Portuguese (PSAS-BR) (Araújo et al. 2024), Italian (Ionio et al. 2024), French (Infante-Gil et al. 2022), Spanish (Costas-Ramón et al. 2023), Chinese (Xu et al. 2021), Persian (Hasanzadeh et al. 2021; Mashayekh-Amiri et al. 2023), and Jordanian Arabic (Hijazi et al. 2024). Additional validations are currently underway in Dutch, German, and other languages.

The COVID-19 pandemic further underscored the importance of assessing postpartum anxiety, as studies have reported a significant rise in maternal anxiety symptoms during this period (Hessami et al. 2020). In response, a 12-item short form was developed for use in global crises (PSAS-RSF-C) and psychometrically evaluated in a UK sample. The English-language version retained the original four-factor structure and demonstrated good model fit and reliability (Silverio et al. 2021). In the same study, the corresponding 12 items were also made available in Italian, French, Chinese, Spanish, and Dutch to increase the international accessibility of the instrument; however, these translated versions were not psychometrically validated in that study. Subsequent evidence suggests that the factor structure may vary across cultural contexts. Specifically, validation of the Persian PSAS-RSF-C among Iranian postpartum women identified a three-factor structure and excluded one item due to a low factor loading (Mashayekh-Amiri and Hasanzadeh 2023).

Beyond the pandemic context, brief psychological instruments have been widely recommended to increase accessibility and improve symptom assessment accuracy (NICE 2018; Silverio et al. 2021). Psychometric validation is essential to ensure reliability and suitability across populations (Keszei et al. 2010). In Brazil, despite high reported rates of postpartum anxiety, existing studies have relied exclusively on general anxiety measures (Loret de Mola et al. 2021; Muller et al. 2022; Segamarchi et al. 2023; Theme-Filha et al. 2024). Therefore, the present study aims to evaluate the psychometric properties of the Brazilian Portuguese version of the PSAS Research Short Form for use in crisis contexts (PSAS-BR-RSF-C) in a sample of Brazilian mothers.

## Materials and methods

### Procedures and sample

The original 51-item PSAS was translated and culturally adapted into Brazilian Portuguese by Araújo et al. (2024), following a systematic process of cross-cultural adaptation and content validation. The present study did not involve a new translation or cross-cultural adaptation process. Instead, the 12 items originally selected by Silverio et al. (2021) for the PSAS-RSF-C were identified within the Brazilian Portuguese version of the 51-item PSAS developed by Araújo et al. (2024). These 12 previously translated and culturally adapted items were then used to compose the PSAS-BR-RSF-C, whose psychometric properties were evaluated in the present study.

A convenience sample of 262 Brazilian mothers aged 18 years or older was recruited through social media platforms between March and November 2022. Data were collected via Qualtrics Survey Software, accessible via smartphones, computers, or tablets. Prior to beginning the survey, participants received detailed information about the study and an informed consent form; consent was confirmed by selecting a checkbox before proceeding. Participants who submitted incomplete questionnaires (*n* = 68) and/or reported having infants with severe illnesses or disabilities (*n* = 5) were excluded, resulting in a final sample of 189 participants.

Inclusion criteria were: (1) being Brazilian; (2) being at least 18 years old; and (3) being the mother of at least one child between 15 days and 12 months of age.

Mothers of infants with severe illnesses or disabilities were excluded because these conditions may involve specific and substantial sources of anxiety related to the infant’s health, prognosis, and caregiving needs, which could differ from postpartum-specific anxiety in the general postpartum population.

No dedicated bot-detection procedures or attention checks were implemented during data collection. IP addresses were monitored to identify potential duplicate submissions, and no duplicate IP addresses were identified.

### Ethical procedures

This study was performed in line with the principles of the Declaration of Helsinki. Approval was granted by the Research Ethics Committee (CEP) of the study’s host institution (CAAE No. 55069122.0000.5336). The ethical aspects proposed by Resolution No. 510/2016 and Resolution No. 466/2012 of the Brazilian National Health Council (CNS) were respected. All participants provided informed consent electronically before completing the instruments.

### Instruments

#### Sociodemographic questionnaire

Developed exclusively for this study, this questionnaire obtained information on women’s age, marital status, occupational status, level of education, family income, parity, psychopathology history, mode of birth, infant sex, infant age, and COVID-19-related information based on the COCOON International Survey (Loughnan et al. 2022).

#### Postpartum Specific Anxiety Scale – Research Short Form – for use in global crises (PSAS-RSF-C)

This 12-item self-assessment instrument uses a 4-point Likert scale. The four-factor structure encompasses: (1) Maternal Competence and Attachment Anxieties (items 1–3); (2) Infant Safety and Welfare Anxieties (items 4–6); (3) Practical Infant Care Anxieties (items 7–9); and (4) Psychosocial Adjustment to Motherhood (items 10–12). Higher scores indicate greater anxiety symptomatology; a score of ≥ 26 indicates a clinically significant level. The original PSAS-RSF-C demonstrated good reliability (McDonald’s ω = 0.87) (Silverio et al. 2021). The Brazilian Portuguese version of the original 51-item PSAS also demonstrated good internal consistency (Cronbach’s α = 0.90) (Araújo et al. 2025).

#### Edinburgh Postnatal Depression Scale (EPDS)

This 10-item self-assessment scale measures depressive symptoms in the past seven days. A cut-off of ≥ 10 is used for probable postpartum depression (Cox et al. 1987). The Brazilian version demonstrated good internal consistency (Cronbach’s α = 0.87) (Santos et al. 2007). In the present study, reliability was McDonald’s ω = 0.89.

#### Generalized Anxiety Disorder scale (GAD-7)

This 7-item self-assessment scale measures anxiety symptoms over the past two weeks on a 0–3 scale (not at all to almost every day) (Spitzer et al. 2006). Total scores range from 0 to 21; anxiety is classified as minimal (0–4), mild (5–9), moderate (10–14), or severe (15–21). The Brazilian version showed good reliability (Cronbach’s α = 0.91) (Moreno et al. 2017). In this study, McDonald’s ω = 0.89.

#### Self-Compassion Scale (SCS)

This 26-item scale assesses self-compassion through three positive components (self-kindness, common humanity, and mindfulness) and three negative components (self-judgment, isolation, and over-identification), rated on a 5-point Likert scale (Neff 2003). For convergent validity, the 13 items comprising the negative components were summed to derive a self-criticism score ranging from 13 to 65, with higher scores indicating greater self-criticism (Brenner et al. 2017; Muris and Otgaar 2020). No established clinical cutoff score is available for this index. Internal consistency for the Brazilian validation was α = 0.92 (Souza and Hutz 2016). In the present study, the self-criticism score yielded McDonald’s ω = 0.91.

### Data analysis

Data were stored in SPSS (version 28.0) and analyzed using JASP (version 0.98.1). Prior to all analyses, variable distribution was verified using the Shapiro–Wilk test (*p* ≤ .05 indicating non-normality). Results indicated a non-parametric sample; appropriate methods were applied accordingly. Descriptive analyses were conducted for categorical variables, including means, standard deviations, and binomial tests.

Confirmatory factor analysis (CFA) assessed the adequacy of both a single general factor and the four-factor structure proposed by Silverio et al. (2021). Given the ordinal nature of the PSAS-BR-RSF-C response categories, all 12 items were specified as ordered categorical variables, and the CFA was based on a polychoric correlation matrix. CFA was conducted using the Diagonally Weighted Least Squares (DWLS) estimator with robust standard errors. Model fit was evaluated using the Comparative Fit Index (CFI ≥ 0.95), Tucker–Lewis Index (TLI ≥ 0.95), Root Mean Square Error of Approximation (RMSEA ≤ 0.06), and Standardized Root Mean Square Residual (SRMR ≤ 0.08) (Brown 2006; Hu and Bentler 1999). Reliability was estimated using McDonald’s omega (ω ≥ 0.70 considered acceptable) (Valentini and Damasio 2016). Finally, Spearman correlations examined associations between the PSAS-BR-RSF-C and the GAD-7, EPDS, and SCS to assess convergent validity.

## Results

### Descriptive statistics

The sample comprised 189 Brazilian women aged 18 to 44 years (M = 31.4; SD = 5.5). Most lived in Rio Grande do Sul (*n* = 92; 48.6%), São Paulo (*n* = 32; 16.9%), or Paraná (*n* = 13; 6.9%). The majority were married or in a stable union (84.1%), in an affective relationship (94.1%), employed or self-employed (71.9%), and held graduate (43.9%) or undergraduate (25.4%) degrees, with a family income between R$ 2,000 and R$ 6,000 (36.5%) or above R$ 9,000 (34.9%). A total of 53.4% reported a prior psychopathology diagnosis (*n* = 101), of whom 38% reported anxiety, 31% depression, and 12% comorbid depression and anxiety. This was the first pregnancy for 69.8% of participants (*n* = 132). On average, infants were 5.19 months old (SD = 3.89); 55.9% were male (*n* = 105). Mean scores were: EPDS = 12.03 (SD = 6.17); PSAS-BR-RSF-C = 26.89 (SD = 6.03); GAD-7 = 8.75 (SD = 5.63); SCS self-criticism subscale = 45.21 (SD = 11.92).

### Correlations among PSAS-BR-RSF-C factors

The four latent factors were significantly and positively correlated, with standardized correlations ranging from 0.42 to 0.61 (Table 1). The strongest association was observed between Maternal Competence and Attachment Anxieties and Infant Safety and Welfare Anxieties (*r* = .61), whereas the weakest was between Infant Safety and Welfare Anxieties and Psychosocial Adjustment to Motherhood (*r* = .42).

**Table 1:** Correlations among the PSAS-BR-RSF-C latent factors

Factor	1	2	3	4
1. Maternal Competence and Attachment Anxieties	—			
2. Infant Safety and Welfare Anxieties	0.61*	—		
3. Practical Infant Care Anxieties	0.55*	0.51*	—	
4. Psychosocial Adjustment to Motherhood	0.58*	0.42*	0.49*	—

Values represent standardized latent factor correlations. * *p*<·001

### Confirmatory factor analysis

**Fig. 1:**
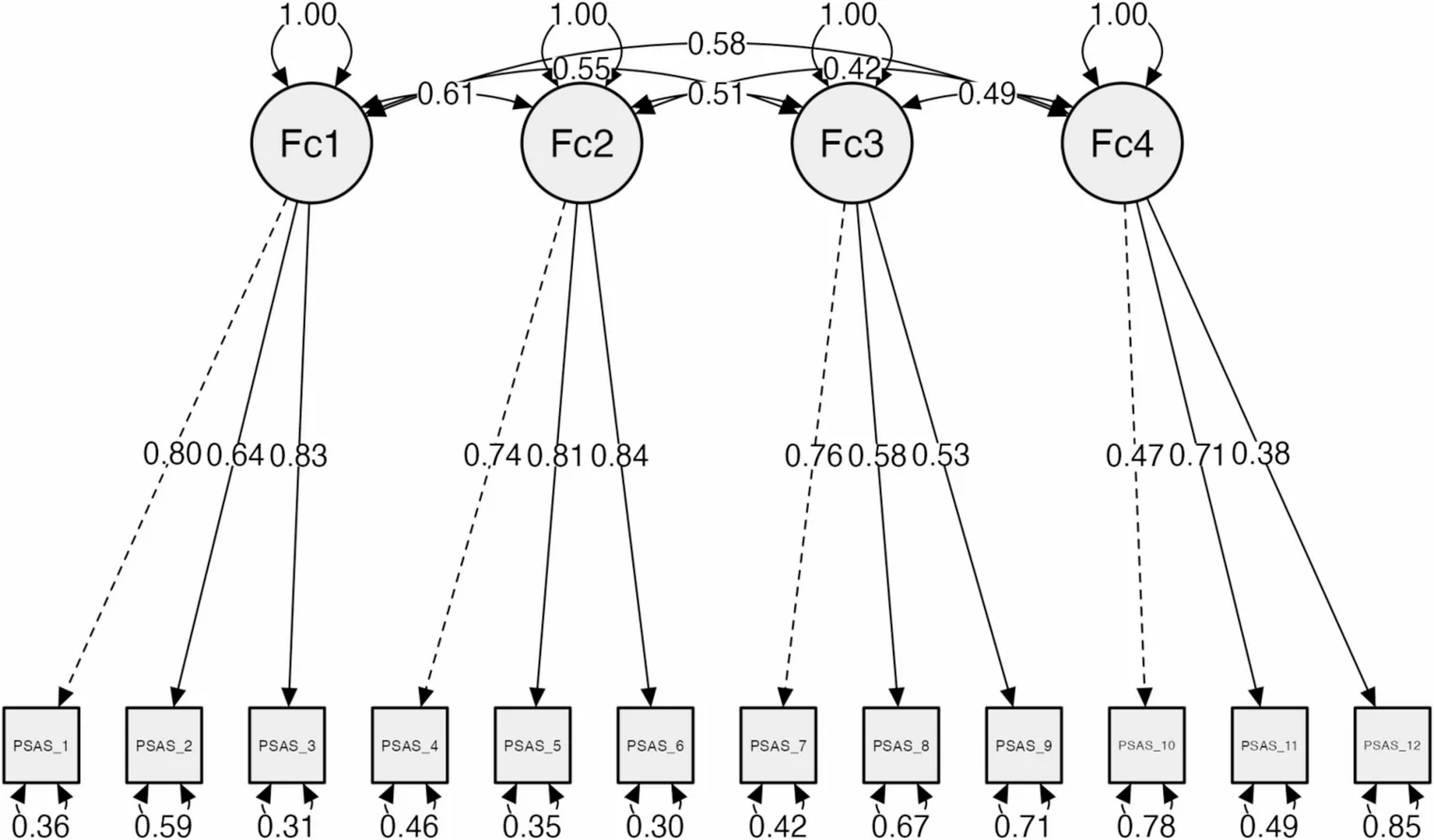
Factor structure of the PSAS-BR-RSF-C

The single-factor model showed inadequate fit, χ²(54) = 180.84, *p* < .001, CFI = 0.913, TLI = 0.893, RMSEA = 0.114, 90% CI [0.096, 0.132], and SRMR = 0.113. In contrast, the hypothesized four-factor model demonstrated substantially better fit, χ²(48) = 79.48, CFI = 0.978, TLI = 0.970, RMSEA = 0.060, 90% CI [0.035, 0.083], and SRMR = 0.077 (Fig. 1). All standardized factor loadings were statistically significant (*p* < .001) and exceeded 0.30, ranging from 0.38 to 0.84. The overall scale demonstrated good internal consistency (McDonald’s ω = 0.85). Factor-level omega coefficients ranged from 0.44 to 0.81 and are presented in Table 2.

**Table 2:** Confirmatory factor analysis with four factors

PSAS-BR-RSF-C items	F1	F2	F3	F4
1. I have had negative thoughts about the relationship with my baby	0.80			
2. I have felt that my baby would be better cared for by someone else.	0.64			
3. I have felt unconfident or incapable of meeting my baby’s basic care needs	0.83			
4. I have worried about my baby being accidentally harmed by someone or something else		0.74		
5. I have repeatedly checked on my sleeping baby		0.81		
6. I have worried that my baby will stop breathing while sleeping		0.84		
7. I have worried about my baby’s milk intake			0.76	
8. I have worried about my baby’s weight			0.58	
9. I have worried about getting my baby into a routine			0.53	
10. *I have felt resentment toward my partner				0.47
11. I have felt tired even after a good amount of rest				0.71
12. *I have worried more about my relationship with my partner than before my baby was born				0.38
McDonald’s Omega (ω)	0.74	0.81	0.62	0.44

### Convergent validity

The PSAS-BR-RSF-C total score was strongly and significantly correlated with GAD-7 (*r* = .52), EPDS (*r* = .61), and self-criticism (SCS; *r* = .52), all *p* < .001, indicating good convergent validity.

## Discussion and conclusions

Postpartum anxiety remains a largely overlooked aspect of maternal mental health, particularly in low- and middle-income countries. This study provides psychometric evidence for a brief and specific measure of postpartum anxiety in Brazil, contributing to the availability of assessment tools for this population. The validation of the PSAS-BR-RSF-C may enable early detection of postpartum anxiety within Brazil’s public health system (SUS), particularly in community-based settings where mental health professionals are scarce. The brevity of the scale makes it suitable for integration into routine postnatal visits.

The CFA supported the hypothesized four-factor structure of the PSAS-BR-RSF-C (Fallon et al. 2016a, b; Silverio et al. 2021), whereas the single-factor model showed inadequate fit, reinforcing the multidimensional nature of postpartum-specific anxiety captured by the scale. All items loaded significantly on their respective factors, with standardized loadings ranging from 0.38 to 0.84. These findings support the distinction among Maternal Competence and Attachment Anxieties, Infant Safety and Welfare Anxieties, Practical Infant Care Anxieties, and Psychosocial Adjustment to Motherhood in the Brazilian version of the scale.

Notably, item 2 of the PSAS-BR-RSF-C (“I have felt that my baby would be better cared for by someone else”) corresponds to item 36 of the original 51-item Brazilian PSAS, which was excluded from the confirmatory factor analysis conducted by Araújo et al. (2025) due to a standardized regression weight below 0.30. In the present study, however, this item showed an adequate standardized factor loading (λ = 0.64) and was therefore retained, suggesting that its performance may vary depending on the item set and sample.

Overall internal consistency across the 12 items was good (ω = 0.85) and closely approximated that reported for the original English PSAS-RSF-C (ω = 0.87; Silverio et al. 2021). However, internal consistency varied across factors. Estimates were satisfactory for Factor 1 (ω = 0.74) and Factor 2 (ω = 0.81), but lower for Factor 3 (ω = 0.62) and particularly Factor 4 (ω = 0.44). In the original English version, the corresponding estimates were 0.88, 0.83, 0.82, and 0.74, respectively (Silverio et al. 2021).

Thus, although the four domains emerged as distinguishable dimensions of postpartum-specific anxiety, the lower internal consistency of Factors 3 and 4 suggests that their current item sets may not assess these specific domains with sufficient precision when interpreted as independent factor scores. Future studies should further examine the item composition of these factors and investigate whether alternative or additional items could provide a more precise assessment of Practical Infant Care Anxieties and Psychosocial Adjustment to Motherhood while preserving their conceptual distinction within the broader construct of postpartum-specific anxiety.

Despite these nuances, the scale showed strong convergent validity with the GAD-7, EPDS, and SCS. This marks a key step in advancing maternal mental health research in Brazil. Although postpartum anxiety lacks formal diagnostic criteria, it is frequently underreported due to inadequate screening tools (Dennis et al. 2017; Zappas et al. 2021; O’Carroll et al. 2022). By providing initial psychometric evidence for a brief postpartum-specific anxiety instrument, this study strengthens research and clinical screening capacity.

Certain methodological limitations should be considered. First, the sample’s lack of socioeconomic diversity — concentrated in the South and Southeast regions, with higher income and education levels — limits generalizability to the broader Brazilian population. Future studies should investigate the instrument’s performance among women with lower income and education. Second, the exclusive use of self-report measures may have introduced response biases, including social desirability and individual differences in the interpretation and reporting of psychological symptoms. In addition, recruitment through social media and online data collection may have introduced self-selection bias, as women with internet access and a willingness or particular interest in participating in research on postpartum mental health may differ systematically from the broader population of Brazilian postpartum women. These factors may limit the representativeness and generalizability of the findings. Third, the scarcity of PSAS-RSF-C psychometric studies restricts cross-cultural comparisons, particularly regarding the stability of its factor structure and factor-level reliability. In the present study, the lower internal consistency observed for Factors 3 and 4, particularly Factor 4, warrants caution when interpreting these factor scores independently and should be further examined in independent Brazilian samples. Fourth, discriminant validity was not assessed, and future studies should examine the extent to which the PSAS-BR-RSF-C distinguishes postpartum-specific anxiety from related constructs such as general anxiety and depressive symptoms. Finally, although IP addresses were monitored for duplicate submissions, no dedicated bot-detection procedures or attention checks were implemented, and therefore automated or inattentive responding cannot be entirely ruled out.

In conclusion, this study represents an important first step in investigating postpartum anxiety among Brazilian women using a validated postpartum-specific instrument. The timing of data collection during COVID-19 adds contextual significance, as this period was marked by heightened psychological distress. As global crises become increasingly frequent, the availability of brief, culturally adapted, and psychometrically sound instruments is crucial. Tools such as the PSAS-BR-RSF-C can enable timely identification of mental health needs in postpartum populations, supporting the development of targeted interventions and helping prevent escalation of maternal anxiety and its negative consequences for maternal and child development (Worrall et al. 2023).

## Electronic Supplementary Material

Below is the link to the electronic supplementary material.

Supplementary Material (PDF 38.9 KB)

## Data Availability

The data that support the findings of this study are not publicly archived due to ethical and privacy constraints arising from the informed consent terms under which they were collected. The raw data are available upon reasonable request to the corresponding author, subject to approval by the Research Ethics Committee (CEP) of Pontifícia Universidade Católica do Rio Grande do Sul (PUCRS).
