# Supplementary material for: Psychometric evaluation of the Brazilian version of the Postpartum Specific Anxiety Scale – Research Short-Form for Global Crises (PSAS-BR-RSF-C)
Source: Arch Womens Ment Health. 2026 Sep 26;29(5):133. doi: 10.1007/s00737-026-01771-6 (PMC13615903; doi:10.1007/s00737-026-01771-6)
Supplement: Supplementary file 1 [file 737_2026_1771_MOESM1_ESM.pdf]

Escala de Ansiedade Específica no Pós-Parto – versão curta (PSAS-BR-RSF-C)

Instruções aos participantes:

Estamos interessados em saber como você está se sentindo desde que teve seu bebê. Por favor, escolha a resposta que mais se aproxima de como você tem se sentido **NOS ÚLTIMOS 7 DIAS**, não apenas como se sente hoje.

Os itens marcados com um asterisco (*) podem não ser aplicáveis a todas as mães. Por exemplo: (*10). Se os itens marcados não se aplicarem a você ou às suas circunstâncias, por favor deixe a resposta do item em branco. Por favor, não tenha pressa e leia cada questão cuidadosamente, selecionando a resposta mais apropriada, cujas opções são as seguintes:

1 = De maneira nenhuma / 2 = Não muito frequentemente /

3 = Frequentemente / 4 = Quase sempre

**Identificação do participante:**	**Idade do bebê em semanas:**	**Data:**

**Número da Pergunta**	**Item da Escala de Ansiedade Específica no Pós-Parto - versão curta**	**1 = De maneira nenhuma**	**2 = Não muito frequentemente**	**3 = Frequentemente**	**4 = Quase Sempre**
1	Tive pensamentos negativos sobre meu relacionamento com meu bebê.				
2	Tenho sentido que meu bebê seria melhor cuidado por outra pessoa.				
3	Tenho me sentido insegura ou incapaz de atender as necessidades básicas de cuidados do meu bebê.				
4	Tenho me preocupado que o meu bebê seja machucado acidentalmente por alguémou por alguma coisa.				
5	Tenho verificado várias vezes meu bebê enquanto ele dorme.				
6	Tenho me preocupado que meu bebê pare de respirar enquanto dorme.				
7	Tenho me preocupado com a ingestão de leite do meu bebê.				
8	Tenho me preocupado com o peso do meu bebê.				
9	Tenho me preocupado em colocar meu bebê numa rotina.				
*10	Tenho sentido mágoa em relação ao meu parceiro.				
11	Tenho me sentido cansada mesmo após um bom repouso.				
*12	Tenho me preocupado mais com meu relacionamento com meu parceiro do queantes do meu bebê nascer.				

* Indica itens que podem não ser aplicáveis a todas as circunstâncias das mães e, portanto, podem ser deixados em branco pela participante.

Gerhardt BC, Zimmer C, Serra J, Souza Torres de Araújo KM, Fallon V, Silverio SA, Arteche AX (2026) Psychometric evaluation of the Brazilian version of the Postpartum Specific Anxiety Scale – Research Short-Form for Global Crises (PSAS-BR-RSF-C). *Arch Womens Ment Health*.
